# Reported Global Avian Influenza Detections Among Humans and Animals During 2013-2022: Comprehensive Review and Analysis of Available Surveillance Data

**DOI:** 10.2196/46383

**Published:** 2023-08-31

**Authors:** Christine M Szablewski, Chelsea Iwamoto, Sonja J Olsen, Carolyn M Greene, Lindsey M Duca, C Todd Davis, Kira C Coggeshall, William W Davis, Gideon O Emukule, Philip L Gould, Alicia M Fry, David E Wentworth, Vivien G Dugan, James C Kile, Eduardo Azziz-Baumgartner

**Affiliations:** 1 Influenza Division National Center for Immunization and Respiratory Diseases United States Centers for Disease Control and Prevention Atlanta, GA United States; 2 Division of Global Health Protection Global Health Center United States Centers for Disease Control and Prevention Atlanta, GA United States

**Keywords:** avian influenza, novel influenza, pandemic influenza, One Health, zoonotic influenza, surveillance

## Abstract

**Background:**

Avian influenza (AI) virus detections occurred frequently in 2022 and continue to pose a health, economic, and food security risk. The most recent global analysis of official reports of animal outbreaks and human infections with all reportable AI viruses was published almost a decade ago. Increased or renewed reports of AI viruses, especially high pathogenicity H5N8 and H5N1 in birds and H5N1, H5N8, and H5N6 in humans globally, have established the need for a comprehensive review of current global AI virus surveillance data to assess the pandemic risk of AI viruses.

**Objective:**

This study aims to provide an analysis of global AI animal outbreak and human case surveillance information from the last decade by describing the circulating virus subtypes, regions and temporal trends in reporting, and country characteristics associated with AI virus outbreak reporting in animals; surveillance and reporting gaps for animals and humans are identified.

**Methods:**

We analyzed AI virus infection reports among animals and humans submitted to animal and public health authorities from January 2013 to June 2022 and compared them with reports from January 2005 to December 2012. A multivariable regression analysis was used to evaluate associations between variables of interest and reported AI virus animal outbreaks.

**Results:**

From 2013 to 2022, 52.2% (95/182) of World Organisation for Animal Health (WOAH) Member Countries identified 34 AI virus subtypes during 21,249 outbreaks. The most frequently reported subtypes were high pathogenicity AI H5N1 (10,079/21,249, 47.43%) and H5N8 (6722/21,249, 31.63%). A total of 10 high pathogenicity AI and 6 low pathogenicity AI virus subtypes were reported to the WOAH for the first time during 2013-2022. AI outbreaks in animals occurred in 26 more Member Countries than reported in the previous 8 years. Decreasing World Bank income classification was significantly associated with decreases in reported AI outbreaks (*P*<.001-.02). Between January 2013 and June 2022, 17/194 (8.8%) World Health Organization (WHO) Member States reported 2000 human AI virus infections of 10 virus subtypes. H7N9 (1568/2000, 78.40%) and H5N1 (254/2000, 12.70%) viruses accounted for the most human infections. As many as 8 of these 17 Member States did not report a human case prior to 2013. Of 1953 human cases with available information, 74.81% (n=1461) had a known animal exposure before onset of illness. The median time from illness onset to the notification posted on the WHO event information site was 15 days (IQR 9-30 days; mean 24 days). Seasonality patterns of animal outbreaks and human infections with AI viruses were very similar, occurred year-round, and peaked during November through May.

**Conclusions:**

Our analysis suggests that AI outbreaks are more frequently reported and geographically widespread than in the past. Global surveillance gaps include inconsistent reporting from all regions and human infection reporting delays. Continued monitoring for AI virus outbreaks in animals and human infections with AI viruses is crucial for pandemic preparedness.

## Introduction

Avian influenza (AI) viruses are a health, economic, and food security risk. While substantial H5, H7, and H9 virus detections in birds have occurred during the past several years, little is known about whether these detections are occurring more frequently or threatening a greater proportion of the world’s poultry, wild birds (especially threatened species), and human populations than in previous years.

AI viruses primarily affect avian species and are grouped as low pathogenicity AI (LPAI) or high pathogenicity AI (HPAI). The difference is based on the ability of the virus to cause mortality and morbidity in poultry or as assessed through genetic sequencing [1]. LPAI causes mild morbidity in poultry but lowers production and may circulate undetected in poultry populations. However, the virus can mutate (specifically H5 and H7 viruses) and become HPAI, which can cause severe disease and mortality in poultry [2]. Migration patterns of asymptomatic reservoirs such as wild waterfowl and shorebirds can lead to the transmission of AI virus to other wild birds and poultry, ultimately contributing to the global spread of AI viruses [3]. Global movement of poultry through trade can further lead to increased AI virus spread [2,4].

AI outbreaks in birds, especially HPAI in poultry, can have large economic implications. A World Bank report from 2008 estimated that global costs attributable to HPAI outbreaks could be as high as 0.7% of global gross domestic product, if AI viruses were to become enzootic throughout the world [5]. Even with control measures, outbreaks of AI viruses continue to occur globally, leading to millions of dead and culled birds, as well as increased chances of virus reassortment events in birds and nonhuman mammals such as pigs and mink [6-8]. HPAI outbreaks can lead to trade restrictions, expensive poultry products, food insecurity, and loss of livelihood [9].

Zoonotically transmitted AI viruses can cause high mortality and may recombine with human influenza A viruses to become more transmissible between humans [10,11]. Efficient and sustained transmission among humans could result in an influenza pandemic with high rates of illness and death [10]. Since 1997, there have been thousands of reports of AI virus infections in humans, most of which have been associated with the H5, H7, and H9 virus lineages [12-14].

A comprehensive review of current global AI virus surveillance information is key to assessing the pandemic risk of AI viruses. This includes reviewing the subtypes of AI viruses circulating during the past decade, their locations, what species are hosting them, and to determine if and why they are increasing in number and spread [15]. The most recent global analysis of official reports of AI outbreaks in birds and human infections with AI viruses was almost a decade ago [16]. In this investigation, contemporary AI virus reports were analyzed among animals and humans from January 2013 to June 2022 and compared with those from 2005 to 2012. AI outbreaks in animals and human AI virus infections were described, detailing the circulating virus subtypes, regions, and temporal trends in reporting animal outbreaks and human cases, and Member Country/Nation characteristics associated with animal AI outbreak reporting; additionally, surveillance and reporting gaps for animals and humans were identified.

## Methods

### Annual and Weekly Records of AI Outbreaks Among Animals

The World Organisation for Animal Health (WOAH) is responsible for improving animal health worldwide. During our timeframe of interest, the WOAH’s Terrestrial Animal Health Code required Member Countries to submit outbreak notifications of all HPAI viruses detected in domestic and wild birds and unusual species (such as mammals), all LPAI viruses of subtypes H5 and H7 detected in poultry, and unusual mortality events among wild birds [1]. The WOAH defines an outbreak as an occurrence of 1 or more cases in a group of animals with a defined epidemiologic relationship, such as a shared environment or common management practices. Each Member Country is responsible for reporting outbreaks to the WOAH. Immediate WOAH notification is not necessary for AI virus subtypes that Member Countries judge endemic [1]. The Food and Agriculture Organization of the United Nations (FAO) publishes monthly reports on all AI virus subtypes including outbreak information for HPAI H5N1 in endemic Member Nations and all LPAI H7N9 viruses [17].

The US Centers for Disease Control and Prevention (CDC) has monitored reports of AI virus outbreaks in animals since 2005. Information about animal outbreaks (in all nonhuman species) reported to the WOAH, FAO, and other official sources (eg, publications and local government websites) was compiled into an AI virus outbreak database that included the number of new outbreaks, by subtype, reported by each Member Country/Nation by report publication year. Starting in 2016, the weekly (based on report publication date) number of newly reported outbreaks, by subtype, from each Member Country/Nation and all animal categories in which that subtype had been reported since 2005 were also included. Animal categories were defined as wild birds (including aquatic and terrestrial waterfowl and shorebirds), zoo/captive birds (including exotic birds), birds in live bird markets, backyard/village birds (poultry such as chickens, ducks, turkeys, and geese), farm/commercial birds (poultry), and nonhuman mammals. All outbreak data are based on the number of outbreak events reported and not on the quantity of animals affected.

Outbreak data from January 1, 2013, to June 30, 2022, were used to determine annual totals for reported outbreaks; the number of Member Countries reporting, by subtype; and the predominant subtype. Subtypes that accounted for the most global outbreaks each year were considered the yearly predominant subtype. Weekly data from January 1, 2016, to June 30, 2022, were used to calculate the total number of outbreaks per subtype per month, as well as the number of newly reported animal categories. A new animal category was defined as a specific animal category, per subtype, reported by a Member Country for the first time since 2005. Results were compared with database information from 2005 to 2012. World Bank data were used to assign region categorization and income classifications to maintain consistency between animal and human health authorities [18]. WOAH data were used to assign the number of individual birds infected and culled [19]. The FAOSTAT was used to assign each Member Nation the percent of global poultry production and total number of annual live-bird imports [20]. The Wetlands International global flyway map was used to determine the number of major migratory flyways passing through each Member Country/Nation [21].

SAS (version 9.4; SAS Institute) was used to conduct univariate analyses of the relationship between independent variables of interest (World Bank income classification, yearly poultry production, total number of flyways through WOAH Member Countries, and total live-bird imports by Member Nations) and the dependent variables of interest (reported animal outbreaks). The regression analysis was restricted to 2013-2020, the years for which FAOSTAT poultry production data were available. Outbreaks from subtypes reported as endemic by a Member Country were excluded from the regression analyses. Variables of interest were assessed in the univariate model and covariates significant at an α level of .10 were included in the multivariable regression model. Relative rates were calculated using a Poisson regression and human population offset. A generalized estimating equation was used to account for correlations within Member Countries during the evaluation period. The final multivariable model was adjusted for World Bank income classification, yearly poultry production, total number of flyways, outbreak year, and total live-bird imports by WOAH Member Countries. Significance for the final model was assigned at *P*≤.05.

### AI Virus Infections Among Humans

The International Health Regulations (IHR) 2005 require Member States to report events of international public health importance to the World Health Organization (WHO) [22]. Human infections with influenza A viruses caused by subtypes that do not circulate in humans (novel) are notifiable [23]. The CDC maintains a line list of human infections caused by novel influenza virus subtypes as reported by the WHO per the IHR (2005) or other official sources (eg, ministries of health, FAO, publications); data were collected by weekly checks of these sources and include Member State of residence or diagnosis, symptom onset and report dates, outcome, exposure information, and reported human-to-human transmission (HTHT, which is defined as infection reported to have been caused by, or to have caused, an infection in another person). We analyzed all human AI virus infection notifications from January 1, 2013 to June 30, 2022.

The number of human cases was calculated by subtype, geographic location, animal exposure, age, reported HTHT, and reported case fatality proportion (rCFP). HTHT ratios (number of cases associated with HTHT divided by all reported infections) and rCFP (deaths noted at report time or in subsequent notifications divided by all reported infections) were calculated by subtype. Reporting lag was calculated as the difference in reported onset date and report date by the WHO in the event information site or other official source. United Nations data were used for population size calculations [24].

### Ethical Considerations

This study was reviewed by the CDC and is consistent with federal law and CDC policy. The study was determined exempt from Institutional Review Board and Institutional Animal Care and Use Committee approval as it uses deidentified data.

## Results

### Annual AI Outbreaks Among Animals

**Figure 1:**
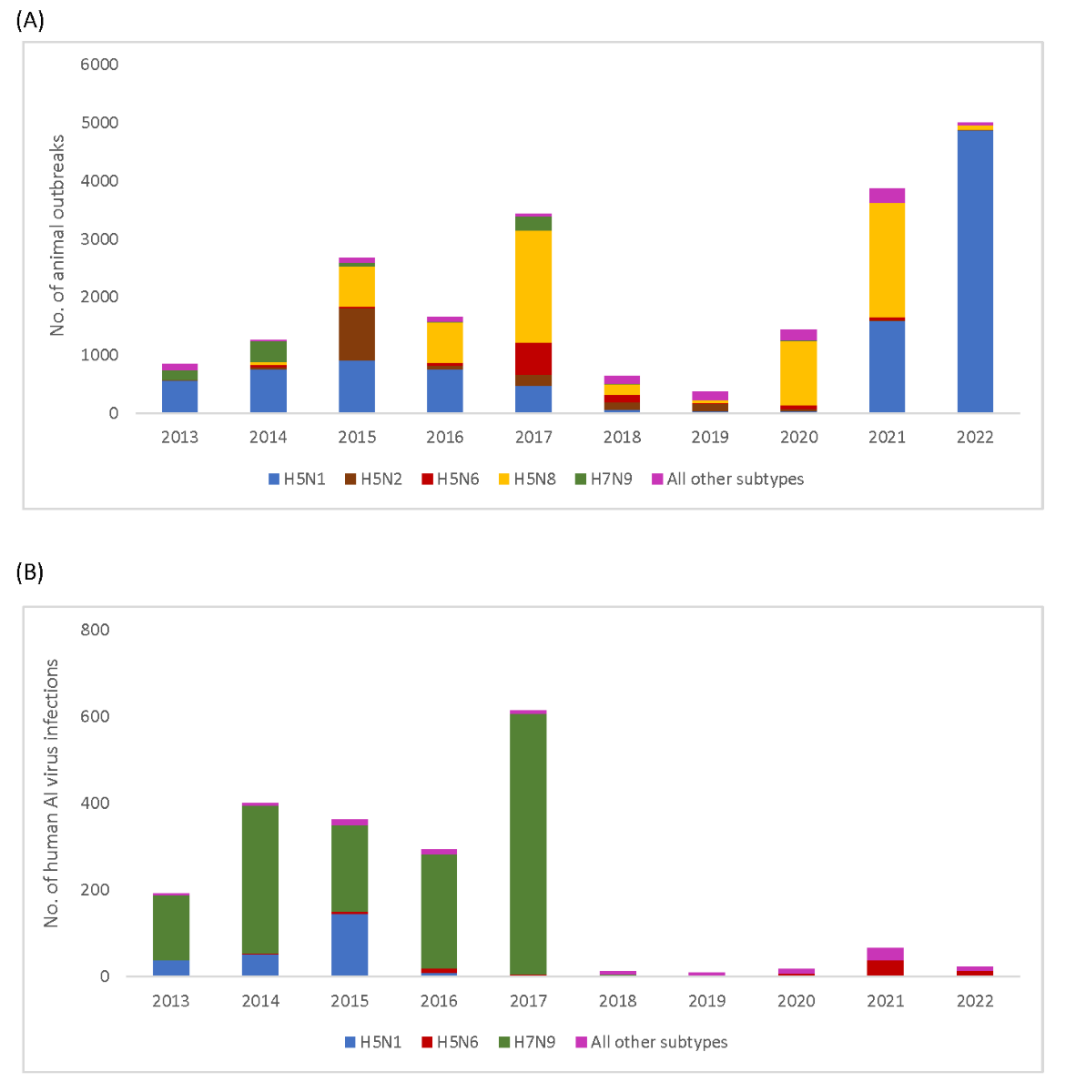
Global animal outbreaks (A) and human infections (B) with avian influenza (AI) virus by subtype, January 2013-June 2022.

**Figure 2:**
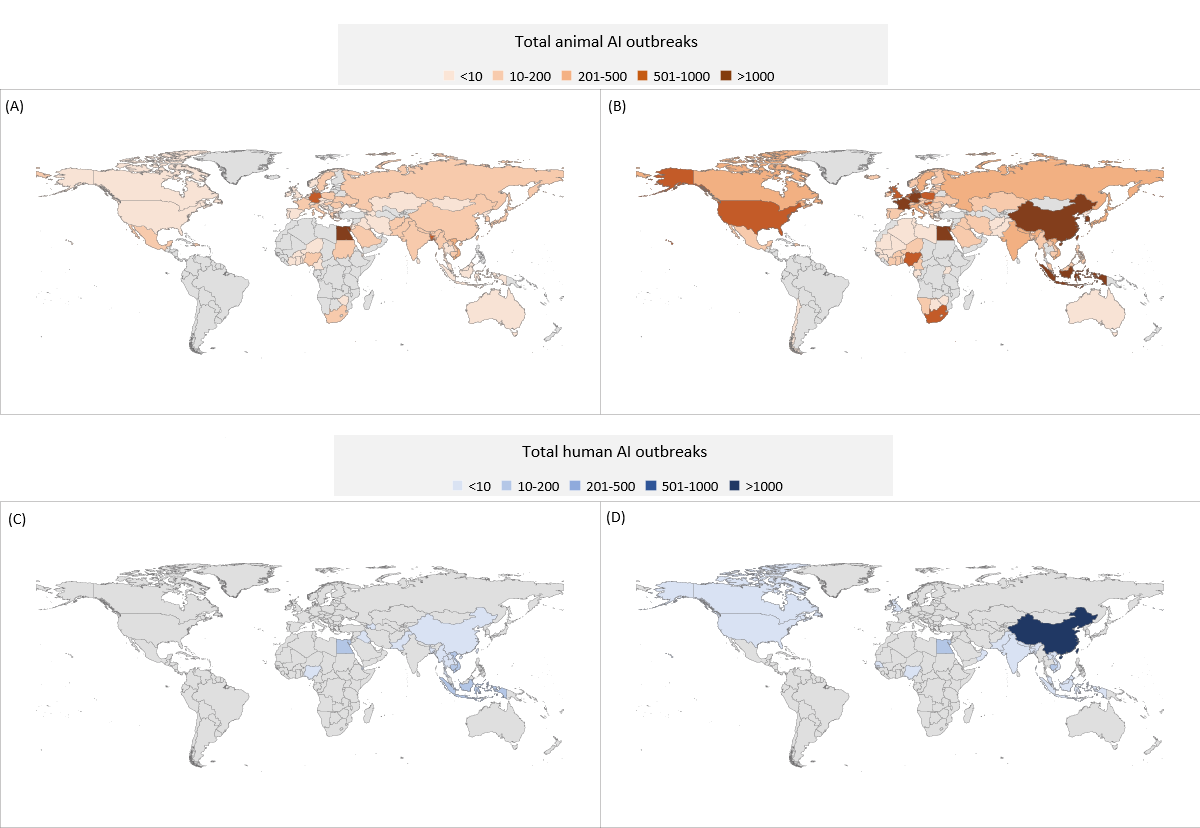
World Organisation for Animal Health Member Countries reporting an animal avian influenza (AI) outbreak from January 2005 to December 2012 (A) and January 2013 to June 2022 (B) and World Health Organization Member States that reported human AI virus infections from January 2005 to December 2012 (C) and January 2013 to June 2022 (D).

Between 2013 and 2022, 95 of 182 WOAH Member Countries (52.2%), which currently produce 87.1% of the world’s poultry [20], reported 21,249 AI virus outbreaks among animals associated with 34 AI virus subtypes (15 HPAI and 19 LPAI); HPAI accounted for 93.39% (n=19,844) of the reported outbreaks (Figure 1 and Table 1); 66% (39/59) of high-income and 31% (9/29) of low-income Member Countries reported an AI virus outbreak during 2013-2022 (Table 2). On average, 42 Member Countries reported AI virus outbreaks each year (65 in 2017 to 20 in 2013); 1 in 4 (26/95) Member Countries first reported AI virus outbreaks among animals in 2013-2022 (Figure 2). All but 6 reporting Member Countries were in the northern hemisphere (Table 2). High-income Member Countries reported the greatest proportion of outbreaks (14,336/21,249, 67.47%; Table 2). Overall, the European and Central Asian regions reported the greatest number of outbreaks (10,550/21,249, 49.65%), whereas Latin American and the Caribbean region reported the fewest number of outbreaks (188/21,249, 0.88%; Table 2 and Figure 2).

**Table 1:** Global avian influenza A virus subtypes reported in animal outbreaks from January 2013 to June 2022.

Subtype		Subtype reported in animals (N=21,249), n (%)
**High pathogenicity avian influenza**		19,844 (93.39)
	H5N1	10,079 (47.43)
	H5N2	1341 (6.31)
	H5N3	41 (0.19)
	H5N4	10 (0.05)
	H5N5	179 (0.84)
	H5N6	926 (4.36)
	H5N8	6722 (31.63)
	H5N9	25 (0.12)
	H5Nx	333 (1.57)
	H7N1	1 (0.00)
	H7N2	2 (0.01)
	H7N3	137 (0.64)
	H7N7	15 (0.07)
	H7N8	1 (0.00)
	H7N9	32 (0.15)
**Low pathogenicity avian influenza**		1405 (6.61)
	H3N1	74 (0.35)
	H5N1	34 (0.16)
	H5N2	211 (0.99)
	H5N3	54 (0.25)
	H5N5	1 (0.00)
	H5N6	5 (0.02)
	H5N8	2 (0.01)
	H5N9	6 (0.03)
	H5Nx	23 (0.11)
	H6Nx	1 (0.00)
	H7N1	11 (0.05)
	H7N2	4 (0.02)
	H7N3	25 (0.12)
	H7N4	3 (0.01)
	H7N6	7 (0.03)
	H7N7	32 (0.15)
	H7N8	8 (0.04)
	H7N9	865 (4.07)
	H7Nx	39 (0.18)

**Table 2:** WOAH^a^ Member Countries reporting avian influenza outbreaks by region and income classification from January 2013 to June 2022.

Region			All WOAH Member Countries^b^ (n=182)		Proportion of all WOAH Member Countries reporting outbreaks^c^ (n=95)		Distribution of reporting Member Countries^d^ (n=95)		Reported outbreaks (n=21,249)
**Hemisphere, n (%)**									
	Northern	154 (84.6)		89 (57.8)		89 (93.7)		20,680 (97.3)	
	Southern	28 (15.4)		6 (21.4)		6 (6.3)		569 (2.7)	
**World Bank region, n (%)**									
	East Asia and Pacific	24 (13.2)		13 (54.2)		13 (13.7)		5815 (27.4)	
	Europe and Central Asia	51 (28.0)		40 (78.4)		40 (42.1)		10,550 (49.6)	
	Latin America and the Caribbean	29 (15.9)		4 (13.8)		4 (4.2)		188 (0.9)	
	Middle East and North Africa	20 (11.0)		10 (50.0)		10 (10.5)		1484 (7.0)	
	North America	2 (1.1)		2 (100.0)		2 (2.1)		1057 (5.0)	
	South Asia	8 (4.4)		6 (75.0)		6 (6.3)		537 (2.5)	
	Sub-Saharan Africa	48 (26.4)		20 (41.7)		20 (21.1)		1618 (7.6)	
**World Bank income classification, n (%)**									
	High income	59 (32.4)		39 (66.1)		39 (41.1)		14,336 (67.5)	
	Upper middle	47 (25.8)		23 (48.9)		23 (24.2)		3564 (16.8)	
	Lower middle	47 (25.8)		24 (51.1)		24 (25.3)		3233 (15.2)	
	Low income	29 (15.9)		9 (31.0)		9 (9.5)		116 (0.5)	
Poultry production^e^,%			99.9		87.1		N/A^f^		N/A
Population^g^			7,770,202,146		6,452,817,271 (83.0)		N/A		N/A

^a^WOAH: World Organisation for Animal Health.

^b^Denominator is all WOAH Member Countries.

^c^Denominator is all WOAH Member Countries in that hemisphere/region/income classification.

^d^Denominator is all WOAH Member Countries that reported an outbreak.

^e^Proportion of world poultry production based on most recently reported data from FAOSTAT for 2020.

^f^N/A: not applicable.

^g^Based on most recently reported data from 2021, Republic of Niue and Cook Islands are excluded from human population totals as data were not available.

A total of 10 HPAI and 6 LPAI virus subtypes were reported for the first time during 2013-2022. As many as 4 HPAI virus subtypes accounted for nearly 90% (n=19,068) of reported animal outbreaks: H5N1 (10,079/21,249, 47.43%), H5N8 (6722/21,249, 31.63%), H5N2 (1341/21,249, 6.31%), and H5N6 (926/21,249, 4.36%); 2 LPAI virus subtypes accounted for 5% (n=1076) of outbreaks: H7N9 (865/21,249, 4.07%) and H5N2 (211/21,249, 0.99%; Table 1). The predominant virus subtype shifted from H5N1 (767/1659, 46.23%, in 2016 to 480/3439, 13.96%, in 2017) to H5N8 (686/1659, 41.35%, in 2016 to 1924/3439, 55.95%, in 2017; Figure 1). H5N8 continued to be the predominant virus subtype in animal outbreaks each year since 2017, except during 2019 and 2022 (Figure 1). Overall, there were approximately 79,709,668 AI virus infections in birds and 323,884,584 birds were culled from 2013 to 2022 per the WOAH.

Results from the multivariable analysis for WOAH Member Countries (Table 3) that reported at least one outbreak from 2013 to 2022 indicated that decreased AI virus outbreak reporting among animals was associated with decreasing country income classification. Compared with high-income Member Countries, on average, upper-middle income countries reported 56% (adjusted relative rate 0.44, 95% CI 0.21-0.90) fewer animal outbreaks, lower-middle income countries reported 82% (adjusted relative rate 0.18, 95% CI 0.09-0.36) fewer animal outbreaks, and low-income countries reported 87% (adjusted relative rate 0.13, 95% CI 0.04-0.43) fewer animal outbreaks. Between January 2013 and June 2022, for every annual poultry production increase of 50 million head, there was a 2% decrease in reported AI outbreaks.

**Table 3:** Multivariable regression model^a^ results for variables of interest associated with reported animal avian influenza outbreaks, January 2013-June 2022.

Independent variable			Crude relative rate		95% CI		*P* value		Adjusted relative rate		95% CI		*P* value
**World Bank income classification**													
	High income	Reference^b^		Reference		Reference		Reference		Reference		Reference	
	Upper-middle income	0.24		0.12-0.45		<.001		0.44		0.21-0.90		.02	
	Lower-middle income	0.12		0.06-0.23		<.001		0.18		0.09-0.36		<.001	
	Low income	0.08		0.03-0.24		<.001		0.13		0.04-0.43		.001	
Annual poultry import (per 50 million head)			1.09		1.00-1.18		.054		0.94		0.85-1.04		.23
Annual poultry production (per 50 million head)			0.98		0.97-0.99		<.001		0.98		0.98-0.99		<.001
**Flyways**													
	1	Reference		Reference		Reference		Reference		Reference		Reference	
	2	0.72		0.35-1.47		.36		1.34		0.72-2.51		.36	
	≥3	0.18		0.09-0.36		<.001		0.67		0.32-1.38		.28	

^a^Poisson distribution with a generalized estimating equation and human population offset adjusted for outbreak year.

^b^No data to be presented here because this is the reference group for the variable.

### Weekly Reports of AI Outbreaks Among Animals

The WOAH received 2485 weekly reports from 2016 to 2022, comprising 15,240 AI virus outbreaks among animals from 91 Member Countries. On average, a median of 13 reports were submitted per week. Taiwan (208/2485, 8.37%) and Germany (176/2485, 7.08%) submitted the most weekly reports. The greatest number of reported animal outbreaks occurred during November 2021-June 2022 (6032/15,240, 39.58%) followed by November 2016-June 2017 (3423/15,240, 22.46%). While outbreaks occurred throughout the year, 87.17% (13,285/15,240) began during November-May (13,024/14,755, 88.27%, in the northern hemisphere Member Countries and 261/485, 53.8%, in the southern hemisphere Member Countries), and onset peaked in January and April in the northern hemisphere and January and November in the southern hemisphere Member Countries (Multimedia Appendix 1). The mean percentage of all outbreaks reported each month that are in a new animal category, for any given member-state, was 14% (range 0%-62%). Of the reports in new animal categories, wild bird (162/439, 36.9%), farm/commercial bird (112/439, 25.5%), and backyard/village bird (105/439, 23.9%) categories comprised most reports. Nonhuman mammals made up the least number of new reports at 1.6% (7/439) of new animal category reports.

### AI Virus Infections Among Humans

During 2013-2022, 17 of WHO’s 194 Member States, which contain more than one-half (4,469,153,194/7,890,000,000, 56.64%) of the world’s population, reported 2000 AI virus infections in humans, which were associated with 10 AI virus subtypes (Figure 1). Both North American region Member States and 40% (4/10) of South Asian region Member States reported a human AI virus infection during 2013-2022 (Table 4). On average, 4 Member States reported human AI virus infections each year (range 2 in 2018 to 7 in 2014). Oman is the only Member State, with a reported human infection, that has never reported an AI outbreak among animals; 8/17 (47%) Member States first reported a human AI virus infection in 2013-2022 (Figure 2).

**Table 4:** WHO^a^ Member States reporting human infection with avian influenza virus by region and income classification (period: January 2013-June 2022).

Region and income classification			All WHO Member States^b^ (n=194)		Proportion of all WHO Member States reporting infections^c^ (n=17)		Distribution of reporting Member States^d^ (n=17)		Reported infections (n=2000)
**Hemisphere, n (%)**									
	Northern	163 (84.0)		17 (10.4)		17 (100)		17 (100)	
	Southern	31 (16.0)		0 (0)		0 (0)		0 (0)	
**World Bank region, n (%)**									
	East Asia and Pacific	32 (16.5)		6 (18.8)		6 (35.3)		1786 (89.3)	
	Europe and Central Asia	50 (25.8)		1 (2.0)		1 (5.9)		1 (0.1)	
	Latin America and the Caribbean	33 (17.0)		0 (0)		0 (0)		0 (0)	
	Middle East and North Africa	20 (10.3)		2 (10.5)		2 (11.8)		195 (9.8)	
	North America	2 (1.0)		2 (100.0)		2 (11.8)		6 (0.3)	
	South Asia	9 (4.6)		4 (50.0)		4 (23.5)		8 (0.4)	
	Sub-Saharan Africa	48 (24.7)		2 (4.2)		2 (11.8)		4 (0.2)	
**World Bank income classification, n (%)**									
	High income	61 (31.4)		4 (6.6)		4 (23.5)		9 (0.5)	
	Upper middle	29 (14.9)		3 (10.3)		3 (17.6)		1743 (87.2)	
	Lower middle	50 (25.8)		10 (20.4)		10 (58.8)		248 (12.4)	
	Low income	55 (28.4)		0 (0)		0 (0)		0 (0)	
Poultry production^e^, %			96.9		67.8		N/A^f^		N/A
Population^g^, n			7,853,022,689		4,469,153,194		N/A		N/A

^a^WHO: World Health Organization.

^b^Denominator is all WHO Member States.

^c^Denominator is all WHO Member States in that hemisphere/region/income classification.

^d^Denominator is all WHO Member States that reported an infection.

^e^Based on most recently reported data from 2020.

^f^N/A: not applicable.

^g^Based on most recently reported data from 2021, Republic of Niue and Cook Islands are excluded from human population totals as data were not available.

H7N9 (1568/2000, 78.40%) and H5N1 (254/2000, 12.70%) viruses accounted for the most human infections (Table 5). China reported the most H7N9 virus infections (1565/1568, 99.81%). Egypt reported the majority of non-H7N9 human AI virus infection cases (194/432, 44.9%), followed by China (170/432, 39.4%). The remaining 15 Member States were responsible for 15.7% (68/432) of cases (Figure 2). Overall, the East Asia and Pacific region reported the greatest number of human cases (1786/2000, 89.30%), followed by the Middle East and North Africa region (195/2000, 9.75%; Table 4). All Member States that reported human infections were in the northern hemisphere; 24% (4/17) were classified as high income and none were classified as low income (Table 4).

**Table 5:** Reported case fatality proportion and known animal exposure for human infections with avian influenza virus (January 2013-June 2022).

Subtype	Cases reported (n=2000), n (%)	Deaths reported (n=446), n (%)	Reported case fatality proportion^a^, (%)	Known animal exposure^b^ (n=1461), n (%)
H3N8	2 (0.1)	0 (0)	0	2 (100)
H5N1	254 (12.7)	89 (20.0)	35.0	219 (86.2)
H5N6	80 (4.0)	27 (6.1)	33.8	70 (87.5)
H5Nx	3 (0.2)	0 (0)	0	3 (100.0)
H7N2	2 (0.1)	0 (0)	0	0 (0)
H7N4	1 (0.1)	0 (0)	0	1 (100.0)
H7N9	1568 (78.4)	326 (73.1)	20.8	1106 (70.5)
H9N2	86 (4.3)	2 (0.4)	2.3	58 (67.4)
H10N3	1 (0.1)	0 (0)	0	0 (0)
H10N8	3 (0.2)	2 (0.4)	66.7	2 (66.7)

^a^Deaths noted at report time or in subsequent notifications divided by all reported infections.

^b^A total of 438 cases had a missing value for the animal exposure variable.

While human AI virus infections occurred throughout the year, 89.65% (1793/2000) had illness onset dates during December-May, with the frequency of onset typically peaking in January (Multimedia Appendix 1). Of the 1953 human infections with available exposure information, 1461 (74.81%) had a known animal exposure prior to illness onset and 101 (5.17%) had no known animal exposure (Table 5). Age and sex information was available for 1989/2000 cases (99.45%); 64.30% (1286/2000) of cases were in males. Most cases were in persons aged 18-64 years (1253/2000, 62.7%). The rCFP varied by virus subtype from 0% (H3N8; 0/2) to 67% (H10N8; 2/3). The overall rCFP was 22.30% (446/2000; Table 5). There were 58/2000 reports of confirmed or possible HTHT (2.90%). The median time from illness onset to the notification posted on the IHR (2005) event information site or other official source was 15 (IQR 9-31; mean 24) days.

## Discussion

### Principal Results and Comparison With Prior Work

Between 2013 and 2022, more AI virus outbreaks in animals were reported by WOAH Member Countries than in the previous 8 years, with 26 Member Countries reporting their first animal AI virus outbreak. From January 2013 to June 2022, there were reports of more than 21,000 AI virus outbreaks in animals and 2000 human infections with AI viruses globally. As many as 10 new HPAI virus subtypes (new hemagglutinin/neuraminidase [HA/NA] combinations) were identified in animals, representing an almost 2-fold increase in the number of subtypes identified during our study period compared with the previous 8 years. This likely reflects broadened viral diversity, increased reporting, and enhanced surveillance. Although there were 34 subtypes reported, only 4 accounted for 89.74% (19,068/21,249) of animal outbreaks; all 4 were of the HPAI H5 lineage. During the study period, the predominant subtype causing animal outbreaks changed from H5N1 to H5N8, for all years but 1. However, in 2022, H5N1 reemerged as the predominant subtype. From 2016 to 2022, about 14% of outbreaks each month occurred in a new animal category with wild birds representing the highest percentage (36.9%, 162/439). This finding suggests that in addition to spreading geographically, AI viruses are also moving into more animal categories. Increases in geographic and animal category reporting could be attributed to increased poultry production and commercial trade [25], increased exposure to wild birds through repeated annual migrations of infected birds, changes in migration patterns because of climate change or land conversion to agricultural production [4,26,27], viral genetic evolution, and improved AI virus awareness and surveillance capacity [28].

Seasonality patterns of animal AI virus outbreaks and human infections with AI viruses were very similar and occurred year-round; the frequency of both peaked during November through May. This finding likely reflects the dominance of reporting from the northern hemisphere countries. The seasonal concurrence of animal and human infections indicates the potential for animal-to-human transmission of AI and highlights the importance of improved monitoring of AI virus subtypes in humans and animals. Animal outbreaks might rise during periods when commercial bird distribution surges in preparation for the winter holidays and lunar new year [16]. This is consistent with our quantitative results which suggest that the risk of human infection increased for those with a known exposure to animals. These epidemic patterns suggest that animal and human health authorities might consider intensifying AI virus infection prevention and control measures immediately before the onset of the November-May increase in AI animal outbreaks [16].

While AI virus outbreaks among animals and human AI virus infections had similar epidemic periods, less than one-half (921/2000, 46.1%) of human cases occurred during years when the same HPAI virus subtype was reported among animals in the same country. In our data set, H9N2 was the third most reported AI virus infection in humans. However, LPAI H9 subtypes are not required to be reported to the WOAH. As human surveillance complements animal surveillance, AI virus detections in humans may serve as signals of AI virus activity among animals. The IHR (2005) stipulates reporting of human AI virus infection within 48-72 hours of identification. Although the time from laboratory detection to reporting was not evaluated (laboratory confirmation date was not routinely reported), the median time of onset to IHR (2005) notification was 15 days with about 26.1% (517/1976) of reports lagging by 30 or more days. Delayed reporting is especially concerning for AI viruses that become capable of efficient HTHT, as significant community transmission may occur by the time of reporting. Timely identification and notification of novel influenza viruses are vital for global pandemic influenza preparedness. The most effective AI virus surveillance would include comprehensive surveillance, inclusive of all subtypes, and timely reporting in domestic and wild birds, humans and, ideally, nonhuman mammals especially swine, as pigs can play a crucial role in novel influenza virus reassortment [6,28].

In our analysis, Member Countries with World Bank income classifications other than high income were less likely to report animal AI virus outbreaks. Latin America and the Caribbean region reported the fewest number of outbreaks. There are published reports of AI virus outbreaks in the Americas, especially Latin America [29]; however, these outbreaks were not reported to the WOAH, and therefore, global surveillance systems that rely on the WOAH reporting mechanism did not capture these outbreaks. A 2022 report from the WOAH noted that HPAI is nationally notifiable in only 73.4% of Member Countries and only 67.5% of Member Countries report having surveillance in place [30]. The lack of established surveillance systems or lack of reporting from these and other Member Countries exposes a gap in global AI virus surveillance. Additionally, the WOAH does not require Member Countries to report enzootic AI [1]. This can lead to routine undercounting of AI virus activity in those Member Countries and shows that while surveillance and reporting may have improved since 2012, there is still room for improvement.

### Limitations

Our study is subject to at least four limitations. First, not all Member Countries/States report AI virus outbreaks among animals or human infections with AI viruses to the WOAH or WHO, respectively, because of inadequate surveillance systems or political and economic concerns about the impact of reporting on poultry trade [28,31]. Further, our multisource data acquisition process may not have identified all cases. Therefore, these reports are likely underestimates. Second, sequencing and clade data were not consistently reported or recorded for animal outbreaks; therefore, genetic relatedness of virus subtypes was not available for this analysis. Third, data collection for the yearly and weekly outbreak and human infection databases occurred manually over the years and is therefore subject to human error. Finally, observed increases in outbreak numbers and reporting Member States/Countries may be an artifact of enhanced surveillance and not necessarily wholly because of an increase in outbreak frequency over time.

### Conclusions

AI virus surveillance from 2013 to 2022 identified 10 new HA/NA combinations of HPAI virus, AI virus in novel animal categories, and 26 more Member Countries reporting animal outbreaks than in the preceding 8 years. Although we are unable to fully quantify the magnitude of each animal outbreak from our data, outbreaks from January 2013 to June 2022 have resulted in the cumulative loss of over 325 million birds. Zoonotic viral transmission from animals to humans poses a continued public health threat because efficient, sustained transmission of AI viruses in humans could trigger the next pandemic [10]. Public and animal health leaders should encourage Member Countries to adhere to international standards put forth by the WOAH and report AI virus events as indicated [1]. Timely notifications from all Member States/Countries of novel influenza virus infections in humans and AI virus outbreaks in animals are central to pandemic preparedness and prevention. It is essential for global AI virus surveillance that WHO Member State and WOAH Member Country health officials ensure that AI virus reports are submitted within the required timeline by international standards. Commonly reported AI virus subtypes in humans were not required to be reported in animals, making One Health collaboration between animal and human surveillance systems even more crucial. Further research to explore causes for AI virus increases in animals might prove beneficial, including evaluation of commercial poultry and swine farm biosecurity, mechanisms of domestic poultry and swine exposure to wild migratory bird populations, laboratory studies to investigate phenotypic characteristics of currently circulating viruses, and the effects of climate and ecological degradation on migration patterns.

## Appendix

Multimedia Appendix 1 Seasonality of global avian influenza animal outbreaks and human avian influenza virus infections, January 2016 to June 2022.

## Abbreviations

AI: avian influenza
CDC: Centers for Disease Control and Prevention
FAO: Food and Agriculture Organization of the United Nations
HA: hemagglutinin
HPAI: high pathogenicity avian influenza
HTHT: human-to-human transmission
IHR (2005): International Health Regulations 2005
LPAI: low pathogenicity avian influenza
NA: neuraminidase
rCFP: reported case fatality proportion
WHO: World Health Organization
WOAH: World Organisation for Animal Health

## Data Availability

The data sets generated during or analyzed in this study may be made available from the corresponding author on reasonable request.
