# Supplementary figures and images for: Reported Global Avian Influenza Detections Among Humans and Animals During 2013-2022: Comprehensive Review and Analysis of Available Surveillance Data
Source: JMIR Public Health Surveill. 2023 Aug 31;9:e46383. doi: 10.2196/46383 (PMC10502594; doi:10.2196/46383)

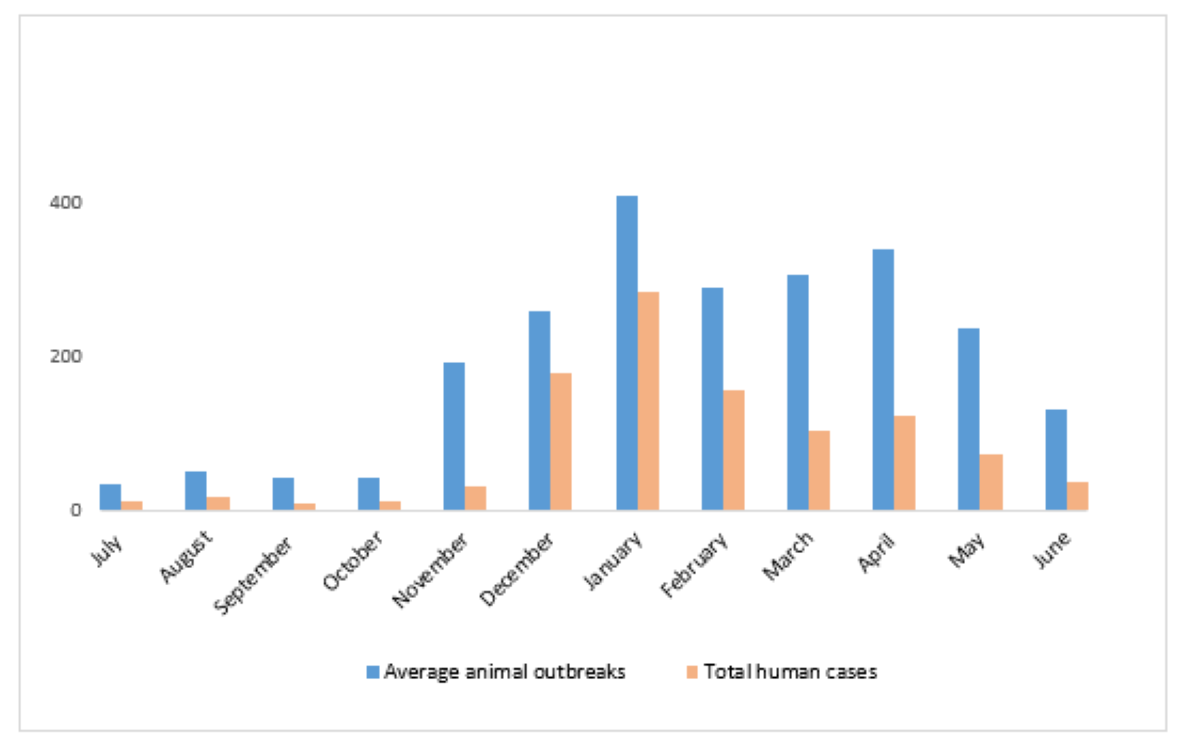

Supplement: Multimedia Appendix 1 [file publichealth_v9i1e46383_app1.png]
